# Sex Differences in the Diagnosis, Management, and Outcomes of Suspected Non-ST-Elevation Acute Coronary Syndromes Meeting Rapid Rule-Out Criteria

**DOI:** 10.3390/jcm12175704

**Published:** 2023-09-01

**Authors:** Ben Cohen, Ruth Tor, Alon Grossman, Ran Kornowski, Avital Porter, David Hasdai

**Affiliations:** 1 Cardiology Department, Rabin Medical Center, Petah Tikva 4941492, Israel; 2 Faculty of Medicine, Tel Aviv University, Tel Aviv 6997801, Israel; 3 Department of Laboratory Medicine, Rabin Medical Center, Petah Tikva 4941492, Israel; 4 Department of Internal Medicine B, Rabin Medical Center, Petah Tikva 4941492, Israel

**Keywords:** acute coronary syndromes, myocardial infarction, troponin, outcomes, sex

## Abstract

(1) Background: patients who meet current rapid rule-out criteria for myocardial infarction (MI) are considered low risk, yet their management remains nebulous, especially among women. We aimed to examine sex differences in the diagnosis, management, and outcomes of patients meeting the rapid rule-out criteria. (2) Methods: by simulating application of the rapid rule-out MI criteria, we analyzed consecutively triaged men and women with suspected NSTE-ACS who had high-sensitivity cardiac troponin T (hs-cTnT) values that met criteria (n = 11,477), in particular, those who were admitted (n = 3775). (3) Results: men constituted ~55% of triaged patients who met the rule-out criteria, whether admitted or discharged. Men were more likely to be admitted (33.7% vs. 31.9%, *p* = 0.04), more commonly with hs-cTnT values between level of detection (LOD, 5 ng/ml) and the 99th percentile (59.4% of all admissions vs. 40.5% for women), whereas women were more likely to be admitted with values < level of blank (LOB, 3 ng/mL; 22.9% vs. 9.2% for men). Thirty-day mortality (1 man and 1 woman) and in-hospital MI (9 men vs. 1 woman) were uncommon among admitted patients, yet resource utilization during 3–4 hospitalization days was substantial for both sexes, with men undergoing coronary angiography (6.8% vs. 2.9%) and revascularization (3.4% vs. 1.1%) more commonly. Long-term survival for both men and women, whether admitted or discharged, was significantly worse for hs-cTnT values between LOD and the 99th percentile, even after adjusting for age and cardiovascular comorbidities. (4) Conclusions: reporting actual hs-cTnT values < 99th percentile allows for better risk stratification, especially for women, possibly closing the sex gap.

## 1. Introduction

Sex bias in the diagnosis and management of acute coronary syndromes (ACS) has been extensively described. Prior studies demonstrated that women with ACS or obstructive coronary artery disease are usually 5 to 10 years older, present with higher-risk baseline clinical characteristics, and eventually have worse outcomes [1,2]. However, they are treated less aggressively in the acute and chronic phases, with a lower tendency to undergo coronary angiography and lower adherence to secondary prevention guidelines [3,4,5].

High-sensitivity cardiac troponins (hs-cTn) are among the major pillars of triage protocols for patients presenting with suspected non-ST-elevation (NSTE) ACS, allowing earlier and more sensitive detection of myocardial ischemia [6]. Sex-related differences in hs-cTn have been described both in the general population and in patients with suspected ACS, yet no sex-specific cut-off levels were suggested to be beneficial in subgroup analyses of studies that tested rapid rule-out criteria using low hs-cTn values [7,8]. Hence, current European and American guidelines have not adopted sex-specific cut-offs for rule-in and rule-out of myocardial infarction (MI) algorithms [9,10].

As we recently showed, very low hs-cTnT values meeting the rapid MI rule-out criteria correlate with favorable in-hospital and long-term prognosis [11]. However, the sex-related incidence of patients meeting these criteria outside the clinical trial setting, their hs-cTn distribution being below the 99th percentile value, and, moreover, their subsequent management and short- and long-term prognosis have not been thoroughly described. Using validated longitudinal data, our study aims to examine the sex differences in the diagnosis, management, and outcomes for this low-risk yet large and resource-consuming population.

## 2. Materials and Methods

This is a subgroup analysis of a large single-center historical prospective cohort study focused on consecutive patients with suspected NSTE-ACS, who had hs-cTnT values that met the rapid rule-out criteria for MI, but were nevertheless admitted. All patients were triaged in the emergency department (ED) of Rabin Medical Center (a tertiary hospital) in Israel between March 2014 through December 2019 [11]. Exclusion criteria included ST-elevation MI or patients referred to the ED for further evaluation, due to abnormal findings in outpatient-based, non-invasive cardiovascular examinations. The local institutional review board approved this study, which was performed in accordance with the Declaration of Helsinki.

As explained in detail in our previous work, we simulated the implementation of the 2020 European Society of Cardiology (ESC) NSTE-ACS rapid rule-out criteria in a large cohort of consecutively triaged men and women (n = 22,717) with suspected NSTE-ACS who had hs-cTnT values that met the rapid rule-out criteria (n = 11,477), in particular, those who were admitted to the hospital (n = 3775) [11]. During this period, the diagnostic algorithm in our medical center applied the 0/3 h algorithm, using hs-cTnT [10]. Hence, values < 99th percentile (14 ng/L), although accurately measured and recorded, were reported qualitatively as “negative” for both medical staff and patients. We therefore analyzed these low hs-cTnT values < 99th percentile, in order to assess which patients would have met the rapid rule-out criteria, had they been implemented in our institution.

As previously described, based on the 2020 ESC NSTE-ACS guidelines, we applied the 0/2 h rapid rule-out algorithm as follows: a single initial value < 5 ng/L (LOD = limit of detection) or an initial value of equal to or greater than 5 ng/L but <14 ng/L (99th percentile value) and an increment less than 4 ng/L in a subsequent test at 2 h, respectively [10,11]. Another important value of the hs-cTnT assay included in our analysis was the limit of blank (LOB = 3 ng/L).

Data were obtained from the Rabin Medical Center electronic records management database and the Rabin Medical Center clinical laboratories database, and mortality data were validated with the Israeli Ministry of Interior database through December 2020. The data obtained included demographic and clinical characteristics, cardiovascular risk factors, co-morbidities, clinical evaluation (e.g., blood tests, stress tests, noninvasive cardiac imaging, and coronary angiography), and revascularization procedures. Lengths of stay at the ED and in hospital were recorded. Discharge diagnoses as well as in-hospital and subsequent mortality were noted.

Our previously published study aimed to compare characteristics and outcomes of patients who met the rapid MI rule-out criteria who were discharged from the ED with those of patients who were admitted [11]. We used our comprehensive data to compare the sex-related demographic, clinical, and laboratory characteristics of patients who met the rapid MI rule-out criteria who were discharged or admitted from the ED. In-hospital resource utilization, diagnosis of MI, and performance of coronary revascularization were followed as well. Moreover, we examined unadjusted and adjusted (age and common baseline cardiovascular risk factors) long-term survival of discharged versus admitted patients.

### 2.1. Study End Points

The primary outcome was a sex-based composite of 30-day mortality and a discharge diagnosis of MI.

The secondary outcomes included length of ED and in-hospital stay, use of non-invasive and invasive diagnostic modalities, and the rate of coronary revascularization procedures. Long-term survival analysis was performed as well.

### 2.2. Statistical Analysis

Continuous data are presented as a median and a 25th–75th interquartile range, and categorical data are presented as proportions (%). The χ^2^ test was used to compare categorical variables. Analysis of variance (ANOVA) was used to compare continuous variables with normal distribution between groups, and the Kruskal–Wallis test was used when normal distribution was not appreciated. The normality of variable distributions was assessed using the Kolmogorov–Smirnov test. Sex-based survival curves were constructed using the Kaplan–Meier method and compared using the log-rank test, with the Tukey–Kramer correction for multiplicity. The Cox proportional hazards model was used to adjust survival for age and common cardiovascular risk factors. All statistical analyses were performed with SAS version 9.4. A *p* value of <0.05 was considered statistically significant.

## 3. Results

**Figure 1:**
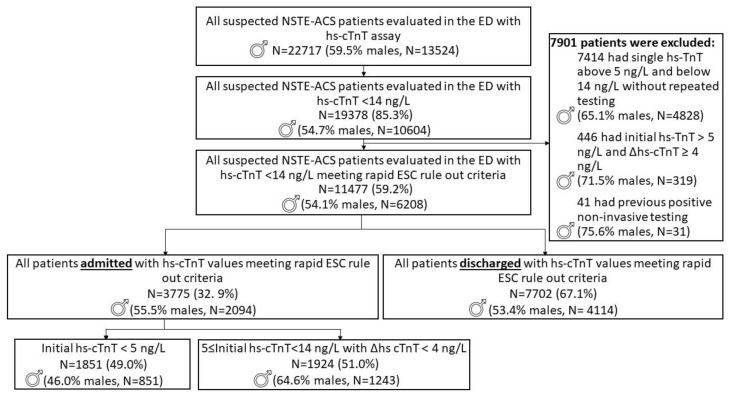
Study cohort flow chart.

During the study period, 22,717 patients were evaluated for suspected NSTE-ACS, accounting for 28,222 ED visits, as some patients visited the ED more than once. Our analysis refers to the index visit to the ED for each patient. Patients were divided by sex and initial hs-cTnT values obtained in the ED. Nearly 85.4% of the obtained values were <99th percentile. Patients who met the rapid MI rule-out criteria constituted 50.5% of all patients undergoing evaluation for suspected NSTE-ACS, and eventually 32.9% of these patients were hospitalized during the study period for suspected NSTE-ACS. In all of the above groups, men constituted the majority, accounting for around 55% of all patients (Figure 1).

Of the 11,477 patients who met the rapid MI rule-out criteria, 6208 (54.1%) were men and 5269 were women (Table 1). When examining the distribution of initial hs-cTnT values by sex among admitted and discharged patients, there were marked differences: 38.4% of men had hs-cTnT values below the LOB, 38.2% had values between the LOB and LOD, and 23.4% had values between the LOD and the 99th percentile, whereas 25.9% of women had values below the LOB, 53.9% had values between the LOB and LOD, and 20.2% had values between the LOD and the 99th percentile (Table 1).

Men were more likely to be admitted than women (*p* = 0.04), but the absolute difference was small (33.7% of men compared with 31.9% of women) (Figure 1 and Table 1). Men were more likely to be admitted with hs-cTnT values between the LOD and the 99th percentile (59.4% of all admissions, compared with 40.5% for women), and women were markedly more likely to be admitted with values below the LOB (22.9% of all admissions, compared with 9.2% for men). Accordingly, men with hs-cTnT values under the LOB were more likely to be discharged than women (53.3% vs. 27.3% of all discharges). For hs-cTnT values between the LOD and the 99th percentile, in contrast, 85.5% of men were admitted compared to 64.0% of women. Among the 1851 patients who were eventually admitted with hs-cTnT values below the LOD, 54.0% were women, and of the 578 patients who were admitted with hs-cTnT values below the LOB, 66.5% were women.

When examining the characteristics of the admitted patients, the median age of men was 5 years lower than that of women (56.0 vs. 61.0 years). Men were more likely to be smokers and to have a prior diagnosis of dyslipidemia and ischemic heart disease, while women were more likely to have hypertension, diabetes mellitus, atrial fibrillation, and hypothyroidism. The prevalence of heart failure and renal failure was low for both sexes (Table 2). In addition, men were more likely to have a second hs-cTnT exam than women, indicating a higher rate of compliance with the guidelines, since staff did not have access to actual hs-cTnT values < 99th percentile. Discharged patients were much younger, with a low rate of comorbidities for both sexes.

The rate of combined primary outcome (a composite of 30-day mortality and a discharge diagnosis of MI) was extremely low in all admitted patients, whether male or female (Table 3). However, numerically, the incidence of MI diagnosis was higher for men (9 men vs. 1 woman). There was one death in each sex groups while in hospital, but neither were attributed to MI.

Although patients admitted with hs-cTnT values meeting the rapid MI rule-out criteria had a relatively low-risk profile, there was considerable use of resources during their hospitalization (Table 3). The length of ED stay was similar between the sexes and across the hs-cTnT categories (5–6 h) as was the median hospital stay (3–4 days). There was a higher tendency to perform coronary computed tomography angiography (CCTA) in women (23.0% of admitted women vs. 20.7% of admitted men), while cardiac radionuclide testing was similarly used (13.4% of admitted women vs. 13.5% of admitted men). As hs-cTnT values increased, the use of cardiac radionuclide testing increased and the use of CCTA decreased in both sexes. Men underwent coronary angiography more commonly (6.8% of admitted men vs. 2.9% of admitted women), and similarly coronary revascularization (3.4% of all men admitted vs. 1.1% of all women admitted). Coronary angiography and revascularization were equally low in both sexes with hs-cTnT values under the LOB (Table 3).

**Figure 2:**
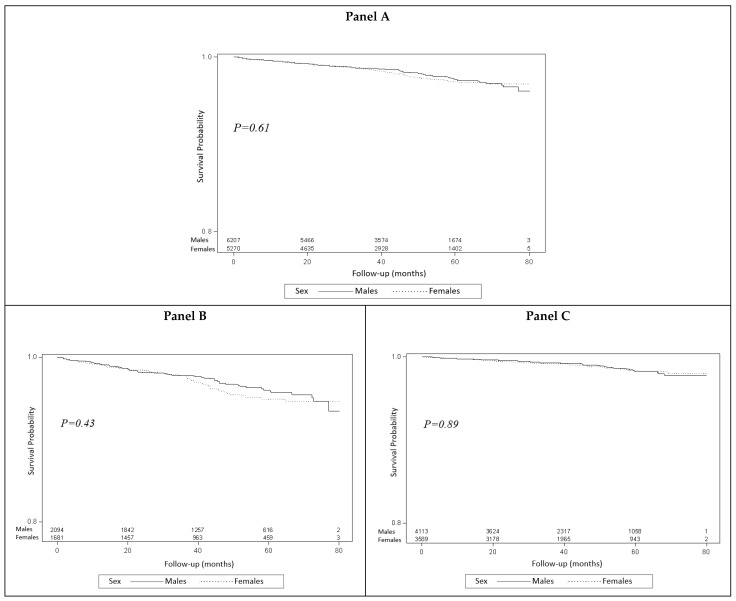
Sex-based survival curves of all patients (Panel **A**), admitted patients (Panel **B**) and discharged patients (Panel **C**) meeting the ESC 0/2 rapid MI rule-out criteria.

When mortality was examined by sex over a follow-up period of 80 months in all patients that met the rapid rule-out criteria, admitted and discharged, sex was not correlated with higher mortality (Figure 2). As baseline characteristics were different between the sexes, we performed a full multivariate model adjusted for age, initial hs-cTnT, and presence of common cardiovascular risk factors (including hypertension, diabetes mellitus, dyslipidemia, atrial fibrillation, hypothyroidism, heart failure, chronic kidney disease, ischemic heart disease, and smoking), yielding no significant difference as well (Supplementary Materials).

**Figure 3:**
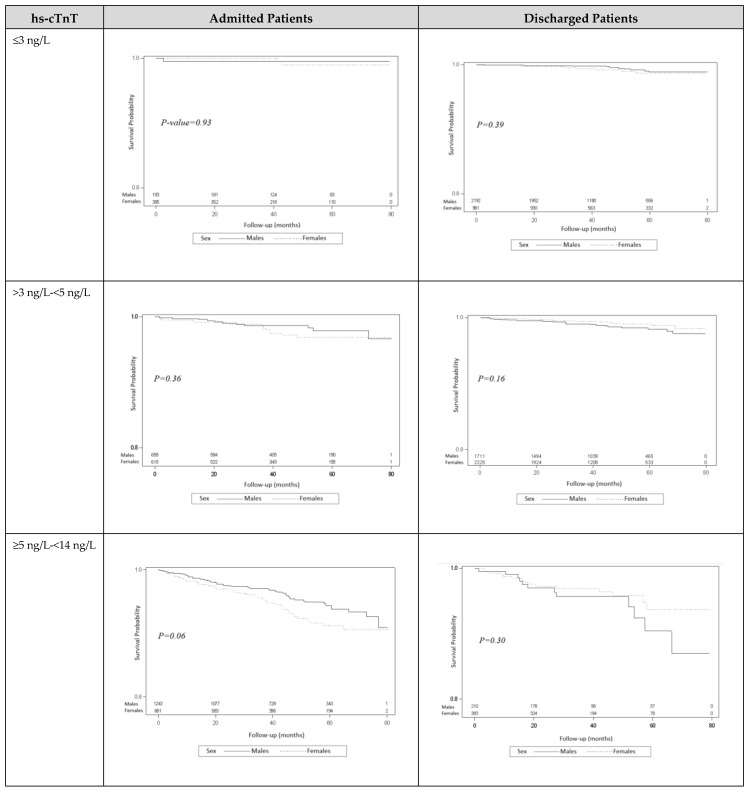
Sex-based survival curves of patients meeting the ESC 0/2 rapid MI rule-out criteria based on admission status and initial Hs-cTnT values.

A survival model based on sex, initial hs-cTnT categories, and whether patients were admitted or discharged demonstrated that the subgroup with hs-cTnT levels between the LOD and the 99th percentile had a higher mortality rate compared with the two other hs-cTnT subgroups (Figure 3). No significant difference in mortality for both sexes was demonstrated in unadjusted models and when performing a full multivariate model, adjusted for age and the presence of common cardiovascular risk factors listed above (Supplementary Materials).

## 4. Discussion

Our study offers a novel insight into the real-world and all-comers’ evaluation of suspected NSTE-ACS patients in the era of hs-cTn-based triage. Specifically, we found differences in demographic and clinical characteristics, biomarker levels, and hospital admission practices based on sex, even among patients with low hs-cTnT values that meet the 2020 ESC rapid MI rule-out criteria. As the application of this algorithm is likely to be increasingly common worldwide, these sex-based differences should be borne in mind when attending to these patients.

The main findings of our study were that: (1) men were more likely than women to present to the ED with suspected NSTE-ACS; (2) patients who met the rapid MI rule-out criteria constituted ~50% of all patients who were evaluated for suspected NSTE-ACS; (3) even at hs-cTnT values < 99th percentile, which are associated with a favorable prognosis, the initial hs-cTnT value was a good prognostic marker for both men and women, with hs-cTnT values below the LOB associated with a much more favorable prognosis, whether patients were admitted or discharged; (4) although altogether men constituted the majority of patients with hs-cTnT values below the LOB and LOD values, this ratio was reversed among inpatients, with a greater tendency to admit women with extremely low hs-cTnT values; (5) men and women who were admitted were subjected to multiple tests over 3–4 hospitalization days, which rarely yielded findings that necessitated revascularization procedures; (6) CCTA was slightly more commonly used in women, whereas the use of coronary angiography as well as revascularization (percutaneous/surgical) were more than two-fold greater in men than women.

Our findings suggest a role for hs-cTnT as a prognostic factor, even in hs-cTnT < 99th percentile for both men and women. Moreover, our study revealed that men with hs-cTnT below the LOD were more frequently discharged from the ED compared to women and vice versa with hs-cTnT above the LOD. We assume that the lower hs-cTnT values in admitted men versus women, whose actual values were not available to the attending medical staff, reflect the fact that routine clinical evaluations frequently fail to assess women’s risk properly. In this respect, we anticipate that the implementation of a rapid rule-out algorithm, with actual hs-cTnT values available to staff, even for values < 99th percentile, will enable better risk reclassification of patients in the ED, especially women. Notably, we did not find a significant difference in short- and long-term mortality between the sexes, whether admitted or discharged, probably reflecting the low-risk characteristics of this population. However, better risk reclassification is expected to improve decisions regarding hospital admissions and resource utilization.

The findings of our study contribute to the understanding of sex differences in suspected NSTE-ACS evaluation and, in particular, rapid hs-cTn-based diagnostic algorithms. Prior reports scrutinizing sex-specific cut-offs for hs-cTn assays concluded that despite evidence that sex-adjusted cut-offs improve sensitivity and specificity in the diagnosis of MI, no prognostic benefit for this practice has been proven so far [12,13]. A previous prospective study that included 1725 healthy individuals and 812 patients with suspected MI demonstrated that age- and sex-tailored cut-off values for hs-cTnT provide better diagnostic information without additional prognostic yield [14].

Our study has several limitations worth mentioning. First, it was a subgroup analysis of a trial designed to examine outcomes and resource utilization in the general population admitted for NSTE-ACS evaluation and meeting the rapid MI rule-out criteria. Second, as explained earlier, it is merely a simulation of the ESC rapid rule-out protocol, based on real hs-cTnT values in the < 99th percentile range, obtained in a very large cohort. Third, the high hospitalization rate (76.4%) among patients with hs-cTnT between the LOD and the 99th percentile may be biased by the requirement for a second hs-cTnT testing to meet the rule-out criteria for our analysis. Indeed, when we examined the hospitalization rate among the 7414 patients who were excluded due to single hs-cTnT testing (Figure 1), it stood at only 31.1%. Finally, as previously mentioned, this study was retrospective, and thus lacked information regarding the precise time of onset of symptoms, electrocardiographic findings, and the staff’s impressions of patients’ complaints (whether suggestive of typical angina or not) [11].

## 5. Conclusions

Our findings highlight that for both sexes there is a high negative predictive value for diagnosing MI applying the rapid rule-out algorithm. These findings also support a policy for both sexes of ED discharge for further evaluation, sparing unnecessary and resource-consuming hospital admissions. By reporting actual hs-cTnT levels for values < 99th percentile, better risk stratification may be achieved for both sexes, resulting in fewer unnecessary hospital admissions and non-invasive and invasive tests. In particular, by recognizing the sex gap in the diagnosis and management of ACS, our findings indicate that access to actual hs-cTnT values < 99th percentile may aid in narrowing this gap.

## Figures and Tables

**Table 1:** Admission vs. discharge incidence by sex and initial hs-cTnT of patients with suspected ACS meeting the ESC 0/2 rapid MI rule-out criteria.

Initial hs-cTnT	Patients, N	Females		Males	
		Discharge, N (%)	Admission, N (%)	Discharge, N (%)	Admission, N (%)
≤3 ng/L	3751	981 (27.3)	385 (22.9)	2192 (53.3)	193 (9.2)
>3 ng/L–<5 ng/L	5209	2224 (62.0)	615 (36.6)	1711 (41.6)	658 (31.4)
≥5 ng/L–<14 ng/L	2517	383 (10.7)	681 (40.5)	210 (5.1)	1243 (59.4)
All	11,477	3588	1681	4113	2094

**Table 2:** Sex-based baseline demographic, laboratory, and clinical characteristics of admitted patients meeting the ESC 0/2 MI rule-out criteria.

	All Admitted Females (N = 1681)	All Discharged Females (N = 3588)	All Admitted Males (N = 2094)	All Discharged Males (N = 4113)
Age (years)	61 (52–70)	39 (31–48)	56 (47–65)	44 (33–55)
**Laboratory**				
hs-cTnT ≤3 ng/L (N, %)	385 (22.9)	981 (27.3)	193 (9.2)	2192 (53.3)
hs-cTnT >3 ng/L–<5 ng/L (N, %)	615 (36.6)	2224 (62.0)	658 (31.4)	1711 (41.6)
hs-cTnT ≥5 ng/L–<14 ng/L (N, %)	681 (40.5)	383 (10.7)	1243 (59.4)	210 (5.1)
Hs-cTnT increment above 4 ng/L in first 24 h (N, %)	21 (1.3)	17 (0.5)	44 (2.1)	12 (0.3)
≥2 hs-cTnT tests performed (N, %)	1091 (64.9)	690 (19.2)	1707 (81.5)	430 (10.5)
Max hs-cTnT value in first 24 h above the 99th percentile (N, %)	54 (3.2)	12 (0.3)	124 (5.9)	9 (0.2)
Creatinine (mg/dL)	0.73 (0.6–0.8)	0.9 (0.8–1.0)	0.92 (0.8–1.0)	0.7 (0.6–0.8)
CRP (mg/dL)	0.38 (0.2–0.9)	0.27 (0.1–0.6)	0.31 (0.2–0.7)	0.36 (0.2–0.8)
LDH (U/L)	382.0 (337.0–445.0)	356 (317–406)	355.0 (314.0–406.0)	356 (310–410)
WBC (K/uL)	7.8 (6.4–9.5)	8.1 (6.7–9.7)	8.2 (6.8–9.8)	7.9 (6.5–9.5)
Hemoglobin (gr/dL)	13.1 (12.2–13.8)	14.9 (14.2–15.6)	14.6 (13.7–15.4)	13.1 (12.3–13.7)
**Vital Signs**				
SBP (mm Hg)	138 (123–152)	130 (120–140)	135 (125–149)	126 (115–139)
DBP (mm Hg)	78 (70–87)	80 (73–89)	82 (74–90)	77 (69–85)
Oxygen Saturation (%)	99 (97–100)	99 (99–99)	98 (97–100)	99.5 (97–100)
Temperature (°C)	36.6 (36.5–36.8)	36.3 (36.3–36.3)	36.6 (36.5–36.8)	36.6 (36.5–36.7)
Pulse (beats/min)	77 (68–87)	78 (68–87)	76 (66–86)	80 (71–89)
**Past Medical History** *				
Hypertension (N, %)	458 (27.3)	56 (1.6)	534 (25.5)	133 (3.2)
Diabetes Mellitus (N, %)	253 (15.1)	36 (1.0)	272 (13.0)	77 (1.9)
Dyslipidemia (N, %)	438 (26.1)	79 (2.2)	585 (27.9)	130 (3.2)
Atrial Fibrillation (N, %)	77 (4.6)	8 (0.2)	69 (3.3)	11 (0.3)
Hypothyroidism (N, %)	132 (7.9)	6 (0.2)	37 (1.8)	98 (2.4)
Heart Failure (N, %)	16 (1.0)	1 (0.03)	18 (0.9)	4 (0.1)
Ischemic Heart Disease (N, %)	148 (8.8)	43 (1.2)	341 (16.3)	29 (0.7)
Smoking (N, %)	82 (4.9)	54 (1.5)	251 (12.0)	18 (0.4)

hs-cTnT= high-sensitivity cardiac troponin; CRP: C-reactive Protein; LDH: lactate dehydrogenase; WBC = white blood cell; SBP: systolic blood pressure; DBP: diastolic blood pressure. * Note that data regarding past medical history of discharged patients may be less accurate because they are based on administrative and ED files rather than actual medical reports.

**Table 3:** Outcomes and resource utilization by sex and initial Hs-cTnT values.

	All Admitted Males (N = 2094)			All Admitted Females (N = 1681)		
	≤3 ng/L	>3 ng/L–<5 ng/L	≥5 ng/L–<14 ng/L	≤3 ng/L	>3 ng/L–<5 ng/L	≥5 ng/L–<14 ng/L
	N = 193	N = 658	N = 1243	N = 385	N = 615	N = 681
**Primary Outcomes**						
Myocardial Infarction (N, %)	1 (0.5)	3 (0.5)	5 (0.4)	0 (0)	0 (0)	1 (0.2)
In-hospital Death (N, %)	0 (0)	0 (0)	1 (0.1)	0 (0)	0 (0)	1 (0.2)
30-day Mortality (N, %)	0 (0)	0 (0)	1 (0.1)	0 (0)	0 (0)	1 (0.2)
Combined Primary Outcome (N, %)	1 (0.5)	3 (0.5)	6 (0.5)	0 (0)	0 (0)	2 (0.3)
**Resource Utilization**						
Emergency Department Stay (hours)	5 (3.07–6.37)	6 (4–7)	5 (4–7)	6 (4–7)	5 (4–7)	6 (4–7)
Hospital Stay (days)	4 (2–5)	3 (2–4)	3 (2–4)	3 (2–4)	3 (2–4)	3 (2–4)
Electrocardiogram Treadmill Test (N, %)	19 (9.8)	32 (4.9)	32 (2.6)	15 (3.9)	9 (1.5)	3 (0.4)
Radionuclide Test (N, %)	10 (5.2)	69 (10.5)	204 (16.4)	24 (6.2)	72 (11.7)	129 (18.9)
Cardiac Computerized Tomography Angiography (N, %)	63 (32.6)	193 (29.3)	178 (14.3)	123 (32.0)	168 (27.3)	96 (14.1)
Coronary Angiography (N, %)	8 (4.2)	34 (5.2)	100 (8.1)	6 (1.6)	18 (2.9)	25 (3.7)
Coronary Revascularization (N, %)	2 (1.0)	28 (4.3)	41 (3.3)	1 (0.3)	10 (1.6)	8 (1.2)

## Data Availability

The data underlying this article will be shared on reasonable request to the corresponding author, Dr. Ben Cohen (bencoh@clalit.org.il).

## Supplementary Materials

The following supporting information can be downloaded at: https://www.mdpi.com/article/10.3390/jcm12175704/s1.
