# Supplementary material for: Sex Differences in the Diagnosis, Management, and Outcomes of Suspected Non-ST-Elevation Acute Coronary Syndromes Meeting Rapid Rule-Out Criteria
Source: J Clin Med. 2023 Sep 1;12(17):5704. doi: 10.3390/jcm12175704 (PMC10488340; doi:10.3390/jcm12175704)
Supplement: Supplementary file 1 [file jcm-12-05704-s001.zip › jcm-2529605-supplementary.pdf]

Sex Differences in the Diagnosis, Management, and Outcomes of Suspected Non-ST-Elevation Acute Coronary Syndromes Meeting Rapid Rule-Out Criteria - Supplements

Ben Cohen , Ruth Tor, Alon Grossman, Ran Kornowski, Avital Porter and David Hasdai

Cox proportional hazards Model for male vs. female for all patients meeting the rapid rule-out MI criteria 2

Cox proportional hazards model for male vs. female for all admitted patients meeting the rapid rule-out MI criteria 3

Cox proportional hazards model for male vs. female for all discharged patients meeting the rapid rule-out MI criteria 4

Cox proportional hazards model for male vs. female for all patients meeting the rapid rule-out MI criteria

Parameter		Pr > ChiSq	Hazard Ratio	95% Hazard Ratio Confidence Limits	
Sex	Female	0.7398	1.047	0.797	1.376
age		<.0001	1.048	1.037	1.060
Decision	Discharge	0.1570	1.276	0.911	1.787
Hypertension	Yes	0.6233	1.100	0.752	1.610
Diabetes	Yes	0.0119	1.640	1.116	2.412
Dyslipidemia	Yes	0.0603	0.690	0.469	1.016
AF	Yes	0.2351	1.398	0.804	2.431
Thyroid disease	Yes	0.0144	1.943	1.142	3.308
Heart failure	Yes	0.1245	1.860	0.843	4.105
CKD	Yes	0.5003	0.672	0.211	2.137
IHD	Yes	0.0423	1.518	1.015	2.270
Smoker	Yes	0.0127	1.920	1.149	3.208
First cTnT result	>3ng/L-<5ng/L	0.0639	1.588	0.974	2.589
First cTnT result	>=5ng/L-14ng/L	<.0001	2.876	1.695	4.879

AF = atrial fibrillation; CKD = chronic kidney disease; IHD = ischemic heart disease.

Cox proportional hazards model for male vs. female for all admitted patients meeting the rapid rule-out MI criteria

Parameter		Pr > ChiSq	Hazard Ratio	95% Hazard Ratio Confidence Limits	
Sex	Female	0.8863	1.028	0.701	1.508
age		<.0001	1.036	1.019	1.053
Decision	Yes	0.5920	1.123	0.735	1.715
Hypertension	Yes	0.0647	1.508	0.975	2.333
Diabetes	Yes	0.0984	0.697	0.454	1.069
Dyslipidemia	Yes	0.1444	1.563	0.858	2.847
AF	Yes	0.0477	1.875	1.006	3.494
Thyroid disease	Yes	0.1692	1.838	0.772	4.376
Heart failure	Yes	0.2015	0.276	0.038	1.992
CKD	Yes	0.0684	1.514	0.969	2.366
IHD	Yes	0.1575	1.525	0.850	2.737
Smoker	>3ng/L-<5ng/L	0.0665	3.084	0.926	10.268
First cTnT result	>=5ng/L-14ng/L	0.0061	5.228	1.603	17.048

AF = atrial fibrillation; CKD = chronic kidney disease; IHD = ischemic heart disease.

Cox proportional hazards model for male vs. female for all discharged patients meeting the rapid rule-out MI criteria

Parameter		Pr > ChiSq	Hazard Ratio	95% Hazard Ratio Confidence Limits	
Sex	Female	0.1614	1.396	0.875	2.227
age		<.0001	1.064	1.045	1.083
Decision	Yes	0.8742	1.077	0.429	2.702
Hypertension	Yes	0.0715	2.280	0.930	5.588
Diabetes	Yes	0.2952	0.612	0.244	1.535
Dyslipidemia	Yes	0.6181	0.673	0.142	3.196
AF	Yes	0.0584	2.780	0.964	8.017
Thyroid disease	Yes	0.2503	3.507	0.413	29.793
Heart failure	Yes	0.1787	2.913	0.613	13.844
CKD	Yes	0.6450	1.273	0.456	3.559
IHD	Yes	0.0402	3.124	1.052	9.276
Smoker	>3ng/L-<5ng/L	0.5892	1.168	0.665	2.050
First cTnT result	>=5ng/L-14ng/L	0.0776	1.902	0.931	3.883

AF = atrial fibrillation; CKD = chronic kidney disease; IHD = ischemic heart disease.
